# Biological quality control for cardiopulmonary exercise testing in multicenter clinical trials

**DOI:** 10.1186/s12890-016-0174-8

**Published:** 2016-01-16

**Authors:** Janos Porszasz, Susan Blonshine, Robert Cao, Heather A. Paden, Richard Casaburi, Harry B. Rossiter

**Affiliations:** Rehabilitation Clinical Trials Center, Los Angeles Biomedical Research Institute at Harbor-UCLA Medical Center, 1124W Carson Street, Building CDCRC, Torrance, CA 90502 USA; TechEd Consultants, Inc., Mason, MI USA; Boehringer Ingelheim Pharmaceuticals Inc., Ridgefield, CT USA; Faculty of Biological Sciences, University of Leeds, Leeds, UK

**Keywords:** Calibration, Treadmill test, Pulmonary gas exchange, Z-score, Precision and accuracy

## Abstract

**Background:**

Precision and accuracy assurance in cardiopulmonary exercise testing (CPET) facilitates multicenter clinical trials by maximizing statistical power and minimizing participant risk. Current guidelines recommend quality control that is largely based on precision at individual testing centers (minimizing test–retest variability). The aim of this study was to establish a multicenter biological quality control (BioQC) method that considers both precision and accuracy in CPET.

**Methods:**

BioQC testing was 6-min treadmill walking at 20 W and 70 W (below the lactate threshold) with healthy non-smoking laboratory staff (15 centers; ~16 months). Measurements were made twice within the initial 4 weeks and quarterly thereafter. Quality control was based on: 1) within-center precision (coefficient of variation [CV] for oxygen uptake [V̇O_2_], carbon dioxide output [V̇CO_2_], and minute ventilation [V̇E] within ±10 %); and 2) a criterion that V̇O_2_ at 20 W and 70 W, and ∆V̇O_2_/∆WR were each within ±10 % predicted. “Failed” BioQC tests (i.e., those outside the predetermined criterion) prompted troubleshooting and repeated measurements. An additional retrospective analysis, using a composite z-score combining both BioQC precision and accuracy of V̇O_2_ at 70 W and ∆V̇O_2_/∆WR, was compared with the other methods.

**Results:**

Of 129 tests (5 to 8 per center), 98 (76 %) were accepted by within-center precision alone. Within-center CV was <9 %, but between-center CV remained high (9.6 to 12.5 %). Only 43 (33 %) tests had all V̇O_2_ measurements within the ±10 % predicted criterion. However, a composite z-score of 0.67 identified 67 (52 %) non-normal outlying tests, exclusion of which coincided with the minimum CV for CPET variables.

**Conclusions:**

Study-wide BioQC using a composite z-score can increase study-wide precision and accuracy, and optimize the design and conduct of multicenter clinical trials involving CPET.

**Trial registration:**

ClinicalTrials.gov identifier: NCT01072396; February 19, 2010.

**Electronic supplementary material:**

The online version of this article (doi:10.1186/s12890-016-0174-8) contains supplementary material, which is available to authorized users.

## Background

Cardiopulmonary exercise testing (CPET) is a non-invasive, sensitive test to evaluate cardiopulmonary (patho-) physiology. CPET assesses the physiological basis of functional capacity and exercise intolerance, and plays a valuable role in diagnosis and clinical decision-making [1]. CPET is also used to test intervention efficacy, e.g., exercise training in cardiovascular disease [2, 3], pulmonary rehabilitation [4–8], and bronchodilator therapy in multicenter trials [9–11]. These applications require strong agreement between consecutively performed tests within and among investigative centers. However, precision (reproducibility, or test–retest variability) and accuracy (trueness) of CPET depend on interactions among: testing equipment variability, calibration, and maintenance; physiological factors; participants’ cooperation, motivation, and effort during testing; and knowledge, skills, and training of laboratory personnel [12]. Most factors cannot be controlled by a simple system calibration, emphasizing the importance of standardization and quality control (QC). Clinicians can rely on trial results only if interpretation is not biased by measurement error [12, 13]. Therefore, assurance of study-wide precision and accuracy has a major impact on the design and conduct of multicenter trials, by maximizing statistical discriminatory power, and minimizing laboratory burden and participant risk.

The American Thoracic Society (ATS)/American College of Chest Physicians (ACCP) [4], American Heart Association [3], and European Respiratory Society (ERS) [7, 12] have published CPET recommendations and standards. These give “best practice” for calibration and QC, and provide typical coefficients of variation (CV) for physiological measurements. ATS/ACCP [4] recommends a biological QC (BioQC) procedure [14] whereby a healthy subject on a stable diet performs regular exercise tests at work rates (WR) below the lactate threshold [12]. As physiologic responses are typically highly reproducible [3, 7, 14–17], the use of healthy individuals performing BioQC can assure reproducibility of the integrated CPET measurement system for patient testing within and between testing centers [18]. Jones and Kane [19] used cycle ergometers and subjects who travelled to each participating center for testing, to demonstrate the efficacy of BioQC to minimize the study-wide CV for oxygen uptake (V̇O_2_; 5.6 %), carbon dioxide output (V̇CO_2_; 8.4 %), and minute ventilation (V̇E; 8.2 %) by excluding the least precise results. Similarly, while Gagnon et al. [20] showed good agreement among five testing centers for V̇O_2,peak_, this approach did not determine the precision and accuracy of measured exercise responses to a standardized protocol. Importantly, the intraclass correlation coefficient at low WR was <0.7 [20], emphasizing the need for QC of the relationship between mechanical and metabolic power output. Brawner et al. [21] used a standardized treadmill protocol, where quality assurance was accepted if at least two of three exercise stages fell within a “target range” for V̇O_2_. Rather than assuring the precision and accuracy of the individual mechanical–metabolic power coupling, the basis of the acceptability criteria (the “target range”) in Brawner et al. [21] was wide, in part due to differences in weights of the volunteers used to develop the normative data.

In this study, we sought to establish new CPET BioQC acceptability criteria that considered both precision and accuracy of the mechanical–metabolic power coupling during a standardized treadmill protocol, and which would be suitable for quality assurance in multicenter studies.

We report the outcome of a precision-based approach to CPET QC in a multicenter trial (ClinicalTrials.gov identifier: NCT01072396) and propose new QC procedures based on both precision and accuracy to minimize variability in multicenter trials. The patient characterization and treatment phases of the parent trial have been reported [22, 23]. Some of the results of this QC study have been previously reported in the form of an abstract [24].

## Methods

### Individuals involved in BioQC procedures

The primary independent Institutional Review Board (IRB), Chesapeake Research Review, Inc. (Columbia, MD, USA) approved the host trial protocol (NCT01072396) for five sites, which could utilize the study’s central IRB, while the remaining 10 participating centers obtained individual IRB/Independent Ethics Committee (IEC) approval (for details see Additional file 1: Table S1). The approved protocol included detailed exercise BioQC procedures in an *Exercise and Quality Control Procedure Manual* in accordance with ATS/ACCP recommendations [4]. During the trial, de-identified BioQC data from a non-smoking, healthy member of the laboratory staff at each center were submitted to a central reader. These laboratory staff members were required to fast and not consume caffeinated drinks for at least 2 h prior to testing, and their age, height, and weight (dressed, wearing shoes) were recorded. This manuscript describes an exempt retrospective study of this de-identified physiologic BioQC data from NCT01072396.

### Equipment used at the study centers

Details of the CPET equipment, software versions, flow sensors, and treadmill details used in the study are shown in Additional file 2: Table S2. The treadmills were run from exercise software, with the exception of one center, where the treadmill was manually adjusted from its own controller using a preapproved procedure.

### Manual of procedures

All centers selected to take part in the trial were provided with an *Exercise and Quality Control Procedure Manual*, which detailed a standardized approach to exercise testing, calibration, and QC. The main objectives of the manual were to: (1) provide information about the available guidelines to promote QC; (2) standardize technical procedures in CPET in order to minimize variation within and between participating centers; and (3) outline and standardize specific procedures involved in the clinical study.

### Staff training, and equipment calibration and verification

Prior to enrolment of patients in the clinical trial, each participating center was visited by a consultant (TechEd Consultants, Inc., Mason, MI, USA) to evaluate and verify the equipment acceptability, and to standardize all QC and test procedures by providing specific training for staff. Training included an initial submaximal incremental exercise test and a BioQC constant work rate test. Details on staff training, and equipment calibration and verification are provided in Additional file 3.

A participating laboratory was only released for patient testing when all CPET technical and equipment performance qualifying criteria were met.

### BioQC procedures

CPET systems were calibrated according to manufacturer’s instructions immediately prior to BioQC testing (see Additional file 3). All centers measured V̇O_2_, V̇CO_2_, and V̇E breath-by-breath via a mouthpiece, and heart rate from the electrocardiogram. The BioQC was a two-stage constant work rate treadmill exercise test. The 18-min protocol consisted of: 3 min of standing rest; 3 min of slow walking (0.8 mph); and 6 min each at 20 W (1.0 mph) and 70 W (1.8 mph). Treadmill grades for each WR were calculated based on participant’s clothed weight [25].

BioQC procedures were carried out at the on-site training visit, within 4 weeks post-training to verify validity of results, and quarterly thereafter for the duration of the study. Systems that required servicing (analyzer replacement, software updates, etc.) underwent an out-of-schedule BioQC test and patient testing was resumed only when the system passed the QC criteria. A maintenance and troubleshooting log was used to record all preventative maintenance activities, as well as faults and repairs (see Additional file 4).

### Accuracy and precision of gas exchange and ventilation measurements

The BioQC tests were analyzed at a central reading site. Physiologic responses were submitted as 10-s bin averages. Steady-state V̇O_2_, V̇CO_2_, and V̇E values were calculated by averaging the last 3 min of exercise at 20 W and 70 W. Acceptability of each BioQC test was established by three methods: the central reader method, the z-score method, and the criterion method.

#### Central reader method

During the multicenter trial, BioQC acceptability was based upon within-center precision. A single central reader compared a BioQC result with the initiation and accumulated quarterly tests within each center. The BioQC was accepted if V̇O_2_, V̇CO_2_, and V̇E were within ±10 % of the initial value based on the expected normal variance [26–29] and ATS/ACCP requirements [4]. This method established precision over time (after an initial accuracy check at the staff-training visit) [4]. A measurement not meeting these criteria was repeated after troubleshooting (see Additional file 4).

#### Z-score method

After trial completion, all BioQC results were assessed en bloc to establish generalizable acceptability criteria. Accuracy and precision of BioQC data were based on “position” and “slope” of the linear relationship between V̇O_2_ and WR [30], as predicted by the following [27]:

1 $$ \overset{.}{\mathrm{V}}{\mathrm{O}}_{\mathtt{2}}=\left(\mathtt{5.8}\times \mathrm{weight}\left[\mathrm{kg}\right]\right)+\mathtt{151}+\left(\mathtt{10.1}\times \mathrm{W}\right) $$

Position was established from % predicted V̇O_2_ at 70 W (V̇O_2,70W_) and slope from % predicted “functional gain” between 20 W and 70 W (V̇O_2,slope_) based on a ΔV̇O_2_/ΔWR of 10.1 mL/min/W [26, 27], which has an established normal range of approximately ±10 % [28, 29]. Thus, accuracy was established by deviation from the predicted value, and precision from the standard deviation (SD) of all BioQC tests. A composite z-score in which position and slope were equally weighted was calculated:

2 $$ \begin{array}{l}\mathrm{z} = \left(\mathrm{ABS}\left(\%\ \mathrm{predicted}\ \overset{.}{\mathrm{V}}{\mathrm{O}}_{2,70\mathrm{W}}-100\right)/\mathrm{S}\mathrm{D}\ \%\ \mathrm{predicted}\ \overset{.}{\mathrm{V}}{\mathrm{O}}_{2,70\mathrm{W}}\right)/2+\\ {}\left(\mathrm{ABS}\left(\%\ \mathrm{predicted}\ {\mathrm{V}\mathrm{O}}_{2,\mathrm{slope}}-100\right)/\mathrm{S}\mathrm{D}\ \%\ \mathrm{predicted}\ {\mathrm{V}\mathrm{O}}_{2,\mathrm{slope}}\right)/2\end{array} $$

where SD is the standard deviation of % predicted for each variable. The Shapiro–Wilk test on the population of % predicted V̇O_2,70W_ and V̇O_2,slope_ measurements was used to identify z-score values associated with a systematic departure from normality (W statistic *P* ≤ 0.05). As the composite z-score criterion is based on % predicted values of all available tests across all centers, it was used to establish the minimum acceptability of both the precision and accuracy of CPET measurements.

#### Criterion method

Both acceptability methods (reader and z-score) were compared with the application of a rigid criterion that no single V̇O_2_ measurement (V̇O_2,20W_, V̇O_2,70W_, and V̇O_2,slope_) should deviate by more than ±10 % from predicted. If any one measurement’s deviation exceeded ±10 % predicted, the test was deemed to have failed QC.

### Statistical analyses

Mean and SD were used to calculate CV for variables within and between centers. Departure from normality was assessed by the Shapiro–Wilk test. For multiple comparisons in one-way analysis of variance (ANOVA), the Student–Newman–Keuls test was used. Statistical significance was accepted if *P* ≤ 0.05. All calculations and statistical analyses were performed using MS Excel (Redmond, WA, USA) and SigmaPlot 12 (Systat, San Jose, CA, USA).

## Results

BioQC laboratory staff were males (*n* = 8; age 41.4 ± 11.4 years, weight 86.6 ± 17.3 kg, body mass index [BMI] 28.0 ± 4.3 kg/m^2^) and females (*n* = 7; age 41.4 ± 8.4 years, weight 61.2 ± 10.8 kg, BMI 23.7 ± 4.9 kg/m^2^). The number of BioQC tests per center varied from five to eight, depending on the length of time the center was active in the trial (16.3 ± 3.1 months [range 10 to 20 months]). This resulted in a total of 129 BioQC tests performed (6.5 ± 1.2 per center), with each center having between 10 and 28 non-repeated pairs of BioQC tests. Overall, therefore, there were 523 unique paired differences of BioQC tests, which were used to establish the distribution properties of the measurements.

### Within-center variability – central reader method

The central reader method accepted 98 (76 %) BioQC tests; 21 (24 %) initial tests required troubleshooting and repetition to meet QC acceptability criteria. At three centers, all tests were accepted. Four centers required one repeat test, two required two repeats, and five required three or more repeats to bring measurements within acceptable CV limits. Thus, across all centers, 31 repeated tests were necessary to bring BioQC measurements within acceptable CV limits (for a study-wide total of 129 tests). Further details on troubleshooting of the CPET systems during the study are provided in Additional file 4.

The within-center mean and variability of accepted BioQC tests are given in Table 1. The most precise variables were V̇O_2_ at 70 W (CV = 5.8 %) and ΔV̇O_2_/ΔWR, which averaged 10.6 ± 0.8 mL/min/W (CV = 5.8 %). The least precise variable was V̇CO_2_ at 20 W (CV = 9.2 %). The assumption that V̇O_2_ at 70 W was below each individual’s lactate threshold was supported by attainment of a steady-state within 6 min.

**Table 1:** Within-center variability of gas exchange and ventilation measurement during treadmill exercise. Measurements were made at work rates of 20 W and 70 W in 98 reader-accepted biological quality control tests

Variable	Work rate (W)	Mean ± SD	Coefficient of variation (%)
V̇O_2_ , L/min	20	0.73 ± 0.15	8.5
	70	1.26 ± 0.17	5.8
V̇CO_2_, L/min	20	0.59 ± 0.12	9.2
	70	1.10 ± 0.12	7.2
V̇E, L/min	20	18.5 ± 4.2	8.3
	70	29.5 ± 4.2	6.3
∆V̇O_2_/∆WR, mL/min/W	∆50	10.6 ± 0.8	5.8

*SD* standard deviation, *V̇O*_*2*_ oxygen uptake, *V̇CO*_*2*_ carbon dioxide output, *V̇E* minute ventilation, *∆V̇O*_*2*_ */∆WR* “functional gain” or the increase in V̇O_2_ per W

### Multicenter precision and accuracy

#### Z-score method

**Fig. 1:**
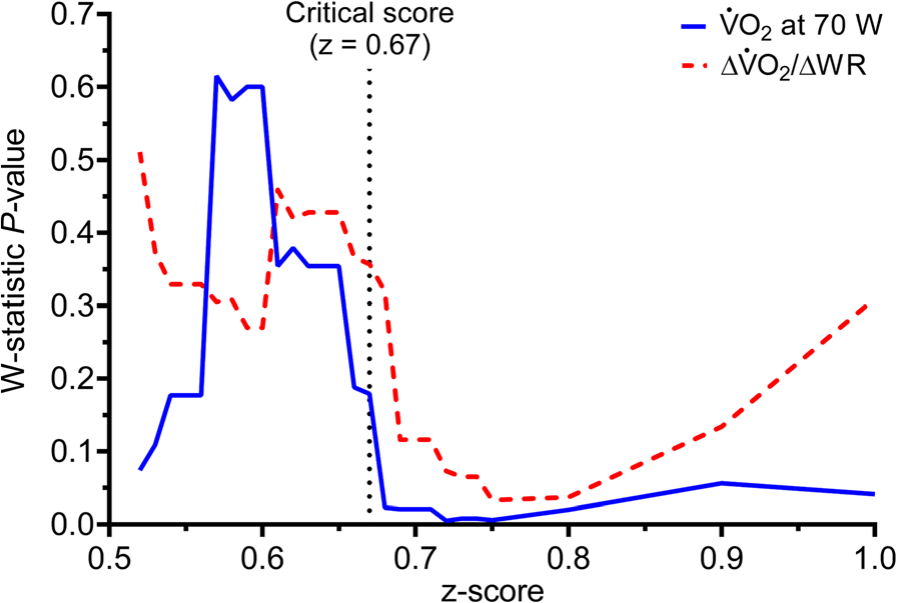
Effect of z-score on normality of oxygen uptake (V̇O_2_) measurement distribution. Normalcy of biological quality control test measurements selected on the basis of a composite z-score. Above a critical z-score of 0.67, the W statistic drops dramatically and the distribution of paired differences becomes non-normal for V̇O_2_ at 70 W

**Fig. 2:**
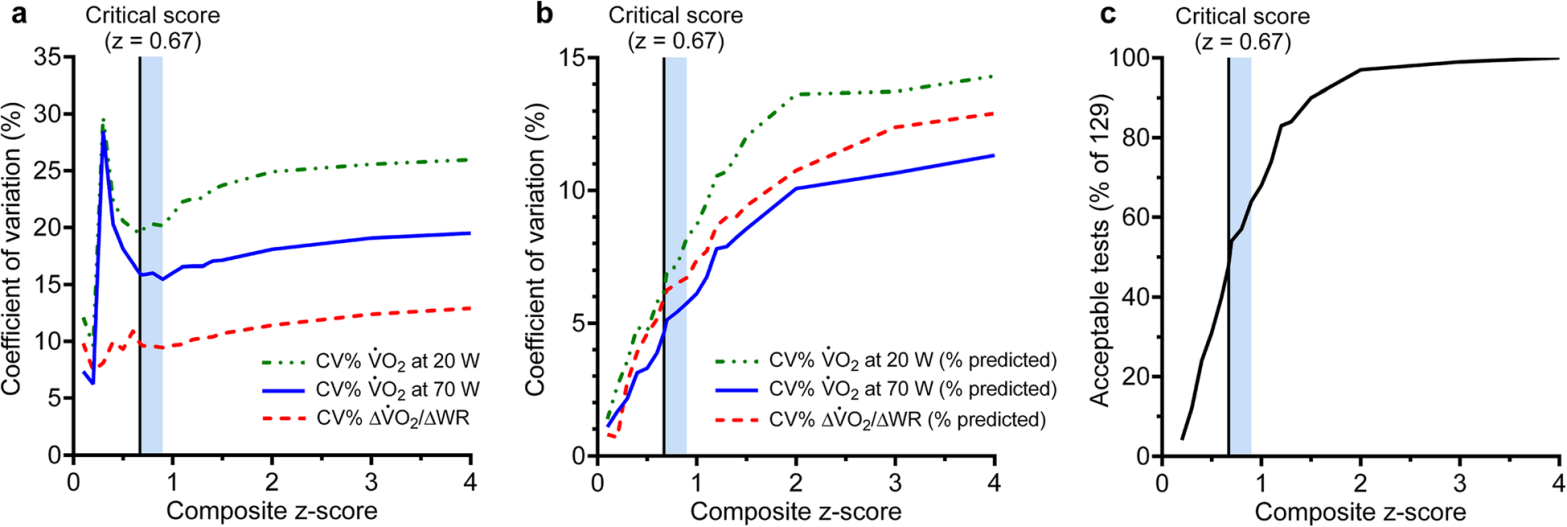
Effect of z = 0.67 cut-off on the CV and number of accepted CPETs. **a** Coefficient of variation (CV) of the absolute oxygen uptake (V̇O_2_) at 20 W, 70 W, and the increase in V̇O_2_ per W (∆V̇O_2_/∆WR), at different z-scores. **b** CV of % predicted V̇O_2_ at 20 W, 70 W, and ∆V̇O_2_/∆WR, at different z-scores. **c** % of acceptable tests (n = 129), at different z-scores. The shaded area is the approximate range of z-scores (0.67 to 0.9) over which absolute measurement CV was minimized (based on panel **a**, and transposed into panels **b** and **c**). The critical z-score is the minimum value for which all measurements are normally distributed. It is noted that the absolute CV (panel **a**) depends on both variability in measurements and differences in weight of individuals performing the biological quality control tests (absolute V̇O_2_ at 20 W and 70 W treadmill walking is dependent on weight). Weight of individuals did not significantly vary during the trial. Despite this, the absolute CV is useful to isolate the z-score range at which the minimum CV occurred (shaded bar). The variability due to measurement differences among centers is better assessed using % predicted values (panel **b**)

Study wide “position” accuracy of CPET measurements in the z-score method was assessed using V̇O_2,70W_ because, of the two steady-state V̇O_2_ measurements, V̇O_2,70W_ showed less variability. Systematic inclusion of non-normal (outlying) BioQC measurements at V̇O_2,70W_ occurred above a z-score of 0.67, while non-normal measurements of ΔV̇O_2_/∆WR occurred above a z-score of 0.75 (Fig. 1). Thus, using a critical z-score of 0.67, 62 (48 %) tests were deemed acceptable. At z = 0.67, multicenter CV for V̇O_2,20W_, V̇O _2,70W_, and ∆V̇O_2_/∆WR were 6.2 %, 4.7 %, and 6.0 %, respectively (Table 2). Accuracy was not different from the criterion method (see below), and close to 100 % predicted in all cases (Table 2). The z-score was the only method for which final selected data for all V̇O_2_ variables were normally distributed (Table 2). Composite z = 0.67 coincided with the lowest CV for absolute measurements in all three variables (Fig. 2a). Systematic increases in V̇O_2_ measurement CV were observed at a z-score of ~0.9 (Fig. 2a) and CV exceeded ATS/ACCP guideline recommendations at a z-score of ~1.0 (Fig. 2b). The CV of % predicted measurements increased with increasing z-score, and z = 0.67 corresponded to a CV in all three variables less than ~6 % (Fig. 2b) incorporating ~50 % of tests (Fig. 2c). Using the composite z-score, a greater number of tests were excluded than using the central reader method (Table 2). Additionally, there was only 53 % agreement in test acceptability between the central reader and composite z-score methods. Overall, precision and accuracy for V̇O_2_ measurements using composite z ≤ 0.67 was greater than the central reader method and very similar to the criterion method, despite substantially more tests than the latter being deemed within acceptable limits.

**Table 2:** Characteristics of variability of oxygen uptake (V̇O_2_) measurements during 129 biological quality control tests, using three different quality control methods

Variable	Work rate (W)	Number (% of all tests)	Mean ± SD (% predicted)	Median (% predicted)	Coefficient of variation (%)	Normality (Shapiro–Wilk)
V̇O_2_ at 20 W						
All tests	20	129 (100)	92.1 ± 12.2	93.5	14.3	Passed
Criterion^a^	20	43 (33)	96.1 ± 5.4	94.8	5.6	*P* < 0.001
Reader^b^	20	98 (76)	91.9 ± 11.5^d^	93.1	12.5	Passed
Composite z-score^c^	20	62 (48)	95.8 ± 5.9	95.0	6.2	Passed
V̇O_2_ at 70 W						
All tests	70	129 (100)	97.5 ± 11.0	97.7	11.3	Passed
Criterion^a^	70	43 (33)	98.4 ± 4.1	98.1	4.2	Passed
Reader^b^	70	98 (76)	97.6 ± 9.4	97.1	9.6	Passed
Composite z-score^c^	70	62 (48)	99.0 ± 4.6	98.4	4.7	Passed
∆V̇O_2_/∆WR						
All tests	∆50	129 (100)	105.7 ± 13.6	105.8	12.9	*P* = 0.006
Criterion^a^	∆50	43 (33)	102.0 ± 5.4	102.5	5.3	Passed
Reader^b^	∆50	98 (76)	106.3 ± 11.4	105.1	10.7	*P* < 0.001
Composite z-score^c^	∆50	62 (48)	103.9 ± 6.2	104.3	6.0	Passed

*SD* standard deviation, *V̇O*_*2*_ oxygen uptake, *V̇CO*_*2*_ carbon dioxide output, *V̇E* minute ventilation, *∆V̇O*_*2*_ */∆WR* “functional gain” or the increase in V̇O_2_ per W

^a^Criterion method was based on V̇O_2,20W_, V̇O_2,70W_ and V̇O_2,slope_ being within ±10 % predicted

^b^Reader method was based on V̇O_2_, V̇CO_2_, and V̇E within ±10 % of the initial value

^c^Composite z-score of 0.67, based on deviation of V̇O_2,70W_ and V̇O_2,slope_ from predicted, with knowledge of SD from all BioQC tests

^d^ *P* < 0.05 Student–Newman–Keuls multiple comparison test within the V̇O_2_ at 20 W

#### Criterion method

When a criterion of ±10 % variability in all V̇O_2_ measurements (% predicted V̇O_2,20W_, V̇O_2,70W_, and V̇O_2,slope_) was applied, only 43 (33 %) tests were acceptable. The criterion method resulted in greater precision (lower CV) and accuracy compared with the central reader method, but was not normally distributed in all variables (Table 2).

### Optimizing precision using distribution analysis of paired differences

**Fig. 3:**
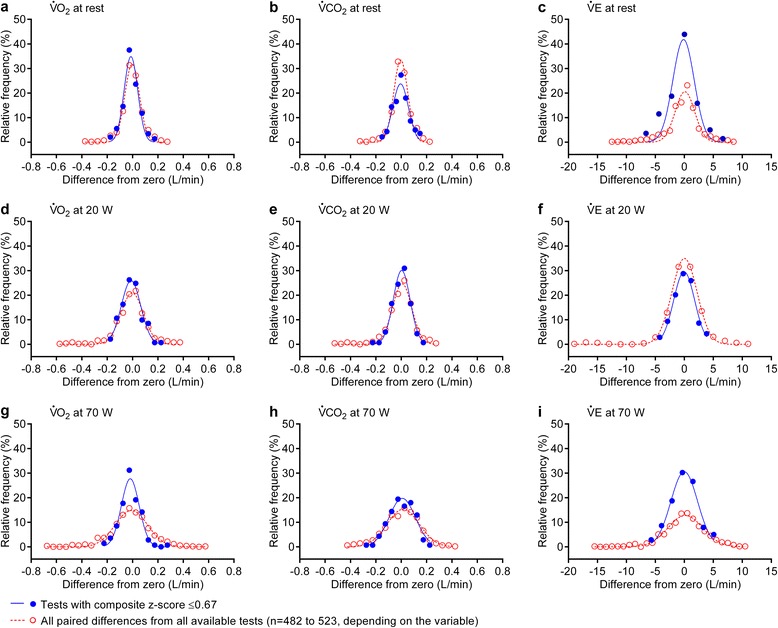
Distribution of relative frequencies of paired differences from zero. Differences from zero for oxygen uptake (V̇O_2_), carbon dioxide output (V̇CO_2_), and minute ventilation (V̇E) at rest (**a**–**c**), 20 W (**d**–**f**), and 70 W (**g**–**i**). None of the variables show normal distribution when all tests are considered (Shapiro–Wilk test, *P* < 0.001). Test selection based on z ≤ 0.67 resulted in all data at each level of exercise (with the exception of V̇E at rest) being normally distributed (Shapiro–Wilk test, *P* > 0.05)

Analysis of 523 paired differences for V̇O_2_, V̇CO_2_, and V̇E is shown in Fig. 3. By including all tests, none of the variables were well described by a Gaussian distribution (Fig. 3, open circles). Whereas, for tests with composite z ≤ 0.67, all gas exchange and ventilation measurements (except V̇E at rest) were distributed normally (Fig. 3, closed circles). A composite z-score based on V̇O_2_ measurement alone was effective in also excluding tests with outlying, non-normal measurements in V̇CO_2_ and V̇E. In all CPET variables, a QC method based on V̇O_2_ precision and accuracy among all centers reduced data variability by ~60 % compared with using no QC method, and by ~50 % compared with the central reader method.

## Discussion

To our knowledge, this is the first study to establish study-wide precision and accuracy for CPET measurements in multicenter trials, based on normalcy of the coupling between mechanical and metabolic power output. We found: 1) the CV of CPET measurements was reduced using the central reader assessment of BioQC exercise tests to minimize within-center variability; 2) a rigid application of a ±10 % predicted V̇O_2_ cut-off criterion excluded 67 % of all measurements, some of which were within the normal distribution of accurate tests; and 3) the application of a composite z-score to identify measurements lying outside normal limits increased the precision and accuracy of multicenter trial CPET measurements by ~50 %.

### Efficacy of CPET QC based on within-center precision

Acceptance rate by the central reader method for all BioQC tests was 76 %. The BioQC process identified measurement errors in 12 of 15 centers before measurements of study patients were made in the parent clinical trial. In six centers, measurement error was resolved by standard troubleshooting approaches, and required only one or two additional BioQC tests to demonstrate resolution. The central reader method excluded outlying tests based on reproducibility, and effectively excluded tests departing from normality. Analysis of all tests showed non-normal distribution, whereas including only tests accepted by the central reader assessment resulted in a data set that was not significantly different from a normal distribution (Table 2). The central reading process reduced within-center CV for gas exchange and ventilation measurements to within the range 5.8 to 9.2 % (Table 1), which is within the range generally accepted for CPET studies [3, 4, 12, 31, 32].

Application of a central BioQC reader, therefore, provided a beneficial addition that reduced measurement variability in the multicenter trial. However, while within-center variability was below the upper limits recommended by ATS/ACCP [4] (~10 %), there remained variability between different centers. The between-center CV for gas exchange measurements of all centrally accepted BioQC tests ranged from 9.6 to 12.5 % (Table 2). This residual between-center variability effectively lessened the benefit of the BioQC method, and weakened the statistical power to demonstrate a given change in CPET outcome measures in the parent clinical trial [23].

### CPET QC based on study-wide precision and accuracy

To our knowledge, only one previous study attempted a QC regimen based on both precision and accuracy [21]. However, the accuracy criterion used was wide (±25 %) because the approach did not account for differences in mechanical power output during treadmill walking at given speed/grade combinations among individuals differing in weight. Therefore, to develop an approach to CPET QC in multicenter trials that included study-wide precision and accuracy in the coupling of mechanical to metabolic power, we applied two different BioQC methods. The first, a simple criterion approach, rigidly excluded BioQC tests in which accuracy of any one V̇O_2_ measurement (V̇O_2,20W_, V̇O_2,70W_, and V̇O_2,slope_) lay outside 90 to 110 % of the predicted value at the calculated power output. This approach appears inherently sensible, in that measurements outside this ±10 % range (roughly based on guideline recommendations [4, 31, 32]) are considered as outlying, and thus excluded. However, a limitation is that a relatively small error in only one variable causes a failing BioQC test, even if all other measurements are within the tolerable limit. The very low acceptability rate of tests across the 15 centers (33 %) makes application of this criterion impractical. Indeed, seven study centers would have been completely excluded from the trial, despite reporting demonstrably accurate measurements based on the retrospective analysis of the normal distribution of the BioQC measurements (Fig. 3). Thus, while the rigid criterion dramatically improved the CV of CPET measurements, it excluded data that were within the normal distribution of measurement variability (e.g*.*, Fig. 1, Table 2).

Therefore, we developed a method that allowed small variability outside the 90 to 110 % range for predicted V̇O_2_, but could successfully identify outlying, non-normal measurements and reduce between-center measurement CV. The composite z-score considered equally, the relative deviation from the mean of “position” (V̇O_2,70W_) and “slope” (ΔV̇O_2_/ΔWR) of the highly predictable relationship between V̇O_2_ and WR. By combining error distribution of these two variables, a small deviation from predicted (i.e., between ~87 % and ~113 %) in one measurement was allowed as long as the other was accurate. We found this method able to strongly predict systematic deviations in non-normal measurements above a composite z-score of 0.67 (based on SD of all tests; Fig. 1). In addition, composite z-score of 0.67 coincided with local minima in CV of absolute V̇O_2_ measurements (Fig. 2a) and resulted in a study-wide CV of ~6 % (Fig. 2b).

Using z = 0.67 we were able to identify that 52 % of the BioQC tests lay outside the normal distribution of V̇O_2_ measurements. While this approach excluded more tests than the central reading method, this combined precision- and accuracy-based method achieved three main benefits. Firstly, it had a strong agreement with the rigid criterion-based approach (84 % agreement between methods). Secondly, it had a relatively high acceptance rate (48 %) without compromising narrow measurement CV compared with the criterion method (Table 2). Lastly, it had a low CV of CPET measurements; ~50 % lower CV than that compared with central precision-based QC approaches that form the basis of guideline recommendations. This latter point is of considerable importance for the design and conduct of multicenter clinical trials with CPET measurement outcomes. By applying a z-score-based BioQC method across all centers, we suggest that measurement variability can be reduced by ~50 %, providing an increase in statistical power to detect changes in CPET measurements. Regular BioQC tests are not onerous and, until a larger BioQC data population is established, any CPET laboratory seeking to implement a QC procedure may simply apply the z-score criterion using equation 2, and the study-wide population SD values established in this study (11.0 % and 13.6 % for V̇O_2,70W_ and V̇O_2,slope_, respectively). While we found the optimal z-score at 0.67, based on distribution normality, the CV of absolute V̇O_2_ measurements remained close to the minimum up to a z-score of ~0.90, corresponding to ~65 % of all tests and a CV for % predicted below ATS/ACCP guidelines (which occurred at z-score ~1.0) [4]. Further research is required to determine more precisely the optimal z-score within the range of ~0.67 to ~0.90 that balances requirements of normality, minimized CV and the number of accepted tests to inform CPET studies. Nevertheless, while using a combined z-score of 0.67 would minimize CPET measurement variability, any power calculations for future clinical trials should also account for the response variability inherent in the clinical population studied. Thus, the combined z-score approach maximizes statistical discriminatory power within multicenter trials and minimizes laboratory testing burden and study participant risk.

### Strategies to minimize measurement variability

We found a relatively high rate of measurement error over 16 months. Importantly, 8 of 15 centers (53 %) required at least one additional validation procedure after an initial failing BioQC test. Each failure triggered a CPET-system troubleshooting process, which included site technicians and central support to identify the error source. Most required involvement of the system manufacturer and eventually led to major equipment service, emphasizing the need for regular maintenance.

In addition, this justifies the recommendation for frequent and rigorously evaluated QC methods in order to prevent large unexplained variability in CPET measurements. The BioQC process used here also identified equipment error prior to equipment failure, and allowed centers to address failing components of CPET systems before trial-related measurements were scheduled. Overall, our results support the view that systematic BioQC is needed to achieve satisfactorily accurate and precise data in multicenter trials employing CPET.

### Limitations

A potential limitation relates to the accurate estimation of treadmill WR. External WR is calculated considering a subject’s weight [33]; but does not account for the inertia associated with body movements while walking [25]. These inertial components increase with weight, which may reduce the accuracy of calculated WR and predicted V̇O_2_ value upon which BioQC is based. A similar phenomenon occurs in cycle ergometry, where V̇O_2_ is influenced by pedaling frequency. However, a change in treadmill speed between 20 W and 70 W (1.0 mph to 1.8 mph) requires an obligatory cadence increase and thus variable internal work; in cycling, cadence can be effectively controlled [34, 35]. One solution would be to recruit BioQC subjects who are similar in weight to potential trial patients. Another solution would be to use calculations for treadmill WR that incorporate kinetic energy (mv^2^) instead of momentum (mv).

Another limitation is that we used an equation to predict metabolic rate that was originally developed for cycle ergometry. However, within the speed range used in this study, measured and predicted metabolic rates show strong agreement [25]. V̇O_2_ prediction was based on a ΔV̇O_2_/ΔWR of 10.1 mL/min/W [26, 27]. A range of studies support this value, e.g., 10.2 ± 1.0 mL/min/W [28] and 9.9 ± 0.7 mL/min/W [29], although it is recognized that a greater value may be seen in endurance-trained individuals [26]. While, in this study we found that ΔV̇O_2_/ΔWR averaged 10.6 mL/min/W, this mean was derived from only 15 individuals who performed the BioQC and was within the normal range. We found that post hoc adjustment of the target ΔV̇O_2_/ΔWR between 10.1 mL/min/W and 10.6 mL/min/W excluded only one additional BioQC test and had no effect on the optimal z-score range. Nevertheless, equations to better calculate treadmill WR to improve accuracy of the ΔV̇O_2_/ΔWR target, or using exercise modalities such as cycling where WR can be better controlled, would likely further improve precision and accuracy provided by the composite z-score BioQC method.

The BioQC method relies on the attainment of steady-state metabolic responses below the lactate threshold at 70 W. This may require verification by an additional incremental exercise test for non-invasive lactate threshold estimation, and/or the use of a lower WR for less aerobically fit or smaller individuals.

The QC method assesses instrumental measurement precision and accuracy (as opposed to physiologic variability) from submaximal steady-state CPET measurements, because the variability of predicted values for healthy participants is low within this domain. However, clinical trials typically assess both submaximal and maximal values from CPET measurements. Therefore, the instrumental measurement precision determined in this study may not necessarily reflect the instrumental precision of peak measurements, where breathing frequency is greater, and the response times of the gas analyzers become increasingly important. Nevertheless, quality assurance of the integrated CPET measurement system linking the mechanical and metabolic power output within the submaximal domain should also contribute to improving assurance of multicenter instrumental precision and accuracy of maximal CPET measurements.

## Conclusions

A central precision-based QC procedure for multicenter studies with CPET as an outcome, reduced measurement variability within center, but was not sufficient to assure between-center measurement accuracy. Thus, we established the distribution of measurements linking mechanical and metabolic power output during multicenter CPET testing, and used this to develop a composite z-score-based method to assess accuracy and precision of CPET measurements. Based on 129 moderate intensity BioQC exercise tests in healthy laboratory staff across 15 centers and 16 months, we found that a composite z-score of 0.67 was able to detect non-normal (outlying) CPET measurements and trigger CPET system troubleshooting, enabling a reduction of multicenter measurement variability by ~50 %. The measurement distribution and z-score method established in this study may be applied to future multicenter studies where CPET variables are measured. Thus, a study-wide precision- and accuracy-based QC process is required to optimize the design, sample size, and conduct of multicenter clinical trials involving CPET measurements.

### Availability of data and materials

Not applicable.

## Additional files

Additional file 1: Table S1. Independent Ethics Committees and Institutional Review Boards for ClinicalTrial.gov: NCT01072396. (PDF 71 kb) Additional file 2: Table S2. Equipment used at the study centers. (PDF 18 kb) Additional file 3:
**Staff training, and equipment calibration and verification.** (PDF 75 kb) Additional file 4:
**Maintenance and troubleshooting log, and troubleshooting of CPET systems consequent to a failing BioQC test.** (PDF 64 kb)

## Abbreviations

ATS: American Thoracic Society
ACCP: American College of Chest Physicians
BioQC: Biological quality control
BMI: Body mass index
V̇CO_2_: Carbon dioxide output
CPET: Cardiopulmonary exercise testing
CV: Coefficient of variation
ERS: European Respiratory Society
IRB: Institutional Review Board
mv^2^: Kinetic energy
VE: Minute ventilation
mv: Momentum
V̇O_2_: Oxygen uptake
QC: Quality control
SD: Standard deviation
WR: Work rate
