# Supplementary material for: Biological quality control for cardiopulmonary exercise testing in multicenter clinical trials
Source: BMC Pulm Med. 2016 Jan 16;16:13. doi: 10.1186/s12890-016-0174-8 (PMC4715309; doi:10.1186/s12890-016-0174-8)
Supplement: Additional file 1: Table S1. — Independent Ethics Committees and Institutional Review Boards for ClinicalTrial.gov: NCT01072396. (PDF 71 kb) [file 12890_2016_174_MOESM1_ESM.pdf]

Table S1 Institutional Review Boards and Independent Ethics Committees for ClinicalTrial.gov: NCT01072396

No. of Centers	Institutional Review Board/Independent Ethics Committee
5	Chesapeake Research Review, Inc., 6940 Columbia Gateway Drive, Suite 110, Columbia, MD 21046-3430, USA
1	John F. Wolf, MD. Human Subjects Committee, Los Angeles Biomedical Research Institute at Harbor UCLA Medical Center, 1124 W. Carson St., Torrance, CA 90502, USA
1	Partners Human Research Committee, Human Research Office, 116 Huntington Avenue, Suite 1002, Boston, MA 02116, USA
1	Western Institutional Review Board (WIRB), 3535 Seventh Avenue SW, Olympia, Washington 98502-5010, USA
1	Springfield Committee for Research Involving Human Subjects, 801 North Rutledge Street, PO Box 19616, Springfield, IL 62794-9616, USA
1	Trustees of Dartmouth College Dartmouth Hitchcock Medical Center Committee for the Protection of Human Subjects, 63 South Main Street, HB 6254, Hanover, NH 03755, USA
1	Saint Frances Hospital and Medical Center, 114 Woodland Street, Hartford, CT 06105, USA
1	McGill University Health Center, Research Ethics Office, 3650 St. Urbain Street, Montreal, QC H2X 2P4, Canada
1	Comité d’Éthique de la Recherche, Institut Universitaire de Cardiologie et de Pneumologie de Québec, 2725 Chemin, Sainte-Foy, Québec, QC G1V 4G5, Canada
1	Health Sciences and Affiliated Teaching Hospitals Research Ethics Board, Queen’s University, Fleming Hall/Jemmett Wing, 3rd Floor, 78 Fifth Field Company Lane Kingston, ON K7L 3N6, Canada
1	CHUM Research Ethics Committee, Cooper Building, Mezzanine 2, 3981 Boulevard St-Laurent, Montreal, QC H2W 1Y5, Canada
