# Supplementary material for: Biological quality control for cardiopulmonary exercise testing in multicenter clinical trials
Source: BMC Pulm Med. 2016 Jan 16;16:13. doi: 10.1186/s12890-016-0174-8 (PMC4715309; doi:10.1186/s12890-016-0174-8)
Supplement: Additional file 2: Table S2. — Equipment used at the study centers. (PDF 18 kb) [file 12890_2016_174_MOESM2_ESM.pdf]

Table S2 Equipment used at the study centers

Center	CPET equipment	Software version	Flow sensor	Treadmill
1	SensorMedics[a]	Vmax™ Spectra 12-7[a]	Mass flow sensor	GE Marquette Series 2000[b]
2	Medical Graphics® Ultima™[c]	7.0.0.26	preVent® flow sensor[c]	Trackmaster® TMX425[d]
3	SensorMedics[a]	Vmax™ Spectra 12-3A[a]	Mass flow sensor	Trackmaster® TMX425C[d]
4	Medical Graphics® Ultima™[c]	6.4	preVent® flow sensor[c]	Trackmaster® TMX425C[d]
5	SensorMedics[a]	Vmax™ Encore 20-7[a]	Mass flow sensor	Trackmaster® TMX425[d]
6	Medical Graphics®[c]	6.4.1.44sp4	Pitot tube	Trackmaster® TMX425CP[d]
7	SensorMedics[a]	Vmax™ Encore 20-B[a]	Mass flow sensor	Trackmaster® TMX425[d]
8	SensorMedics[a]	Vmax™ Encore 20-5B[a]	Mass flow sensor	Trackmaster® TMX425[d]
9	SensorMedics[a]	Vmax™ Spectra 12-3A[a]	Mass flow sensor	GE Marquette Series 2000[b]
10	SensorMedics[a]	Vmax™ Encore 21-1A[a]	Mass flow sensor	GE Marquette Series 2000[b]
11	SensorMedics[a]	Vmax™ Legacy 7-2B[a]	Mass flow sensor	GE T-2100[b]
12	Medisoft Expair[e]	1.28.20	Pitot Tube	GE Marquette Series 2000[b]
13	SensorMedics[a]	Vmax™ Spectra 12-2A[a]	Mass flow sensor	Woodway Desmo[f]
14	SensorMedics[a]	Vmax™ Spectra 12-1A[a]	Mass flow sensor	Quinton MedTrack ST55 [g,h]
15	Medical Graphics®[c]	6.4.1.44sp4	preVent® flow sensor[c]	Trackmaster® TMX425[d]

[a]Sensormedics, Yorba Linda, CA, USA.

[b]GE, Fairfield, CT, USA.

[c]Medical Graphics Corporation, St Paul, MN, USA.

[d]Trackmaster, Newton, KS, USA.

[e]Medisoft, Sorinnes, Belgium.

[f]Woodway, Waukesha, WI, USA.

[g]Quinton Instrument Co., Bothell, WA, USA.

[h]The treadmill was manually adjusted from its own controller (a pre-approved procedure).
