# Supplementary material for: Biological quality control for cardiopulmonary exercise testing in multicenter clinical trials
Source: BMC Pulm Med. 2016 Jan 16;16:13. doi: 10.1186/s12890-016-0174-8 (PMC4715309; doi:10.1186/s12890-016-0174-8)
Supplement: Additional file 3: — Staff training, and equipment calibration and verification. (PDF 75 kb) [file 12890_2016_174_MOESM3_ESM.pdf]

Staff training, and equipment calibration and verification

Prior to enrolment of patients in the clinical trial (NCT01072396), each participating center was visited by a consultant (TechEd Consultants, Inc., Mason, MI, USA) to evaluate and verify the equipment acceptability, and to standardize all quality control (QC) and test procedures by providing specific training for staff. A participating laboratory was only released for patient testing when all cardiopulmonary exercise testing (CPET) technical and equipment performance qualifying criteria were met.

The CPET qualifying criteria were as follows. Verification of a valid calibration syringe certification (which was maintained throughout the study). Verification that the CPET gas volume measuring device adhered to American Thoracic Society (ATS)/European Respiratory Society (ERS) standards [1]. Calibration was performed with a 3 L syringe and the recovered volume measurements were required to be within 3% of the expected volume. Linearity of the volume measuring device was subsequently checked as per ATS/ERS recommendations using a 3 L syringe across three flow rates (<2 L/s, 4–6 L/s, and >8 L/s). The gas analyzer qualification was based on: (1) time delay and rise time of the gas analyzers within manufacturer-specified limits; and (2) gas concentration measurements within 0.03% of the certified calibration gas. The accuracy of the gas volume and concentration calibrations were required to be within the specified ranges outlined above, regardless of the make, model, and software version of the CPET equipment used. Volume calibration and gas analyzer calibrations were required immediately prior to any testing.

At each center, treadmill speed and grade were verified once at the beginning of the study. Treadmill calibration was repeated if the treadmill was moved, serviced,

repaired, or replaced, or as part of troubleshooting following a failed biological QC (BioQC) test. Speed and length of the belt were measured using a mark made on the belt and a stopwatch. The number of belt rotations in 1 minute was counted, and then rechecked with an average-sized individual on the treadmill. The treadmill grade was checked by measuring the treadmill height from the ground versus the length of the treadmill. Documentation of each treadmill check was maintained. The quarterly biologic constant work rate (CWR) exercise test also monitored treadmill function.

After all the technical specifications had been met, an incremental exercise test (IET) and a BioQC procedure were completed under supervision. Prior to testing, an adequate warm-up time was allowed for each piece of equipment. For the tests, the weight (wearing clothing and shoes appropriate for walking exercise) of a healthy, nonsmoking member of the laboratory staff was measured and used to calculate the treadmill grade required to achieve target work rates [2,3] at defined speeds. The treadmill IET was performed to verify correct programing of the protocol. This test included: 3 minutes of rest; 3 minutes of slow walking at 0.8 mph and 0% grade; and thereafter, the treadmill speed and grade were adjusted every minute to achieve a 10 W/min step-incremental profile [3]. The IET was stopped at a predetermined submaximal 150 W. The BioQC protocol was CWR exercise at 20 W and 70 W, which were verified to be below the lactate threshold. Inspiratory capacity (IC) was measured serially in both the IET and CWR test, so that the technician could practice the timings of the required maneuvers used in the parent clinical trial. During all tests, Borg scores for breathing difficulty and leg discomfort (Borg CR10 scale) [4] were collected at 2-minute intervals, 30 seconds prior to each IC measurement, according to the host study protocol.

Immediately before any CPET measurements, the ambient temperature, barometric pressure, and relative humidity were verified, and the calibration results of the volume measurement device and gas analyzers were determined to be within the limits specified above. Calibration activities were recorded on a Calibration Report.

BioQC procedures were carried out at the on-site training visit, within 4 weeks post-training to verify validity of results, and quarterly thereafter for the duration of the study.

References

1. Miller MR, Hankinson J, Brusasco V, Burgos F, Casaburi R, Coates A, et al. Standardisation of spirometry. Eur Respir J. 2005;26(2):319-38.
2. Jones NL. Clinical Exercise Testing, 3rd ed. Philadelphia: W.B Saunders; 1988.
3. Porszasz J, Casaburi R, Somfay A, Woodhouse LJ, Whipp BJ. A treadmill ramp protocol using simultaneous changes in speed and grade. Med Sci Sports Exerc. 2003;35(9):1596-603.
4. Mahler DA, Rosiello RA, Harver A, Lentine T, McGovern JF, Daubenspeck JA. Comparison of clinical dyspnea ratings and psychophysical measurements of respiratory sensation in obstructive airway disease. Am Rev Respir Dis. 1987;135(6):1229-33.
