# Supplementary material for: Biological quality control for cardiopulmonary exercise testing in multicenter clinical trials
Source: BMC Pulm Med. 2016 Jan 16;16:13. doi: 10.1186/s12890-016-0174-8 (PMC4715309; doi:10.1186/s12890-016-0174-8)
Supplement: Additional file 4: — Maintenance and troubleshooting log, and troubleshooting of CPET systems consequent to a failing BioQC test. (PDF 64 kb) [file 12890_2016_174_MOESM4_ESM.pdf]

Maintenance and troubleshooting log

A maintenance and troubleshooting log was used to record all preventative maintenance activities, as well as the occurrence of faults and their repairs.

Systems that required servicing (analyzer replacement, software updates, etc.) underwent an out-of-schedule biological quality control (BioQC) test and patient testing was resumed only when the system passed the QC criteria. A Quality Control Log was used to record all QC activities e.g., BioQC and syringe validation of the linearity of the volume measurements. These documents were reviewed by the site monitor as part of the parent clinical trial (NCT01072396).

Troubleshooting of CPET systems consequent to a failing BioQC test

During the study, BioQC tests that resulted in data outside the acceptability criteria prompted an evaluation of the source of error to determine troubleshooting steps. Typically, basic maintenance steps were undertaken on the cardiopulmonary exercise testing (CPET) system to correct a problem, e.g., replacement of the gas sampling line, checking the time delay between the gas and volume sensors, checking the gas analyzer rise times, replacement of the mass-flow sensor, replacement of the calibration gas, or recalibration of the treadmill.

At two centers, diurnal variation or other physiologic factors, such as completing the test after a meal or work-related stress appeared to impact the results. Controlling the environment (ensuring a quiet environment, free of disturbance) during the BioQC tests reduced measurement variability. At one center, the equipment was in a variable temperature environment, which likely impacted the results. Major hardware changes (such as gas sample line pumps and gas

analyzers) were required to improve results in at least five centers. An acceptable BioQC test was then required before further testing of study patients was allowed.

In addition, the BioQC results identified impending hardware failures prior to equipment failure. At one center, a complete gas sample line pump failure occurred after changes in the BioQC results were noted. In most cases, the troubleshooting would have likely been avoided had the manufacturer's recommended maintenance steps been followed.
